# Two mouse models for recoverin-associated autoimmune retinopathy

**Published:** 2010-10-02

**Authors:** Ying Lu, Shirley He, Lin Jia, Naheed W. Khan, John R. Heckenlively

**Affiliations:** Department of Ophthalmology and Visual Sciences Kellogg Eye Center, University of Michigan

## Abstract

**Purpose:**

Recoverin has been demonstrated to be one of the main causative antigenic retinal proteins common in many cases of autoimmune retinopathy (AIR). Strategies for producing two different AIR mouse models associated with anti-recoverin antibodies were tested.

**Methods:**

(1) Six-week-old female B6.MRL-*Fal^lpr^*/J mice (LPR) mice were immunized with recombinant recoverin three times at 2–4 week intervals. (2) Five-month-old Balb/cJ mice were injected with hybridoma cells designed to produce recoverin monoclonal antibodies. Anti-recoverin antibodies were analyzed by immunoblot and enzyme-linked immunosorbent assay (ELISA). Electroretinograms (ERG), histopathologic examination, and flow cytometric analysis were assessed.

**Results:**

High anti-recoverin antibody levels were achieved in both models, accompanied by significantly reduced scotopic and photopic responses on the ERGs. Retinal histology showed swollen cell bodies in the inner nuclear layer in recoverin-immunized LPR mice, while photoreceptor and outer nuclear layer swelling was observed in recoverin hybridoma cells injected into balb/cJ mice. Glial fibrillary acidic protein (GFAP) staining detected a marked increase of Müller cells and astrocyte reactive gliosis in both mouse models. Rhodopsin and S-opsin staining was similar to controls, while decreased numbers of bipolar cells were observed in both models. Complement component C1q and C3 deposits increased upon immunohistopathologic retinal staining in both models, while increased numbers of CD4+ and CD68+ cells from retinas were found upon flow cytometric analysis.

**Conclusions:**

These two models had similar pathology in the retina, indicating the retinal antigens to recoverin antibody set off pathologic events that include leukocyte invasion, complement deposition, reactive gliosis in the retina, and selective retinal degeneration of inner nuclear layer neurons. These two AIR mouse models will allow for detailed pathologic investigation and testing of protein antigens associated with human AIR and can be used to test treatments. It is important to note that, since most AIR patients have multiple anti-retinal antibodies, it will be possible to study which antibodies are pathologic and which have no retinal pathologic effects. These models can also serve as an important research resource for studying the pathophysiology of specific retinal proteins by creating autoantibodies, which potentially will give a better understanding of retinal protein interactions.

## Introduction

Autoimmune retinopathy (AIR) is an ophthalmologic disease characterized by sudden and progressive loss of vision in association with circulating anti-retinal autoantibodies (ARAs). The condition may occur as a primary autoimmune condition or in association with various cancers. Three forms have been reported over the last 15–20 years, including cancer-associated retinopathy (CAR), melanoma-associated retinopathy (MAR), and non-paraneoplastic AIR (npAIR) [1-3]. Complicating the understanding of AIR are cases that have a secondary complication occurring in other disorders such as retinitis pigmentosa or birdshot retinopathy, meaning there can be several clinical manifestations related to anti-retinal antibodies [4,5]. Most AIR patients do not have a previous history of visual problems or night blindness until they suddenly develop the onset of photopsias, followed by other symptoms such as night blindness, scotomata, and visual field loss. No discernible retinal changes are initially seen upon fundus examination. However, standardized electroretinography (ERG) will show abnormal responses. Many patients also have negative waveforms on dark-adapted bright flash testing, which, along with the constellation of the above symptoms and findings, is a hallmark of the disease [6]. Most patients do not have pigmentary deposits, but exhibit a diffuse hypopigmented atrophic retinopathy. Autoimmune retinopathy typically occurs without any sign of anterior or posterior uveitis (cells or flare upon biomicroscopy). A few AIR cases may show retinal vascular sheathing [7,8]. While AIR patients typically have these common clinical features, no uniform set of ARAs have been found to be circulating in these patients; typically AIR patients have three to six different ARAs found on immunoblots, and the number of bands and intensity of staining is stronger in affected patients compared to normal control samples. In general, AIR is treatable with immunosuppression therapies [1,9]. Currently, most immunoreactive bands on west blot represent unknown retinal proteins. Many of the known antigenic retinal proteins with pathologic associations have not been widely investigated for lack of a good animal model system.

Autoantibodies against various retinal proteins, including recoverin, α-enolase, transducin-α, S-arrestin, heat shock proteins, carbonic anhydrase II (CA II), aldolase A, aldolase C, TULP-1, and others have been found in patients with AIR [1,10,11]. We identified anti-recoverin and six other antibodies in several patients with MAR using proteomic analysis [1,10,11]. For about a decade, it was believed that recoverin, often referred to as the CAR antigen, was the sole autoantigen involved in the development of CAR. Anti-recoverin antibodies have been used to diagnose CAR, but other cancer-associated antibodies have also been found [3,10,12-14]. Patients with CAR have loss of both rods and cones, indicating widespread retinal dysfunction [15,16].

The protein recoverin is found in photoreceptor and retinal bipolar cells and is a Ca^2+^-binding protein that plays a regulatory role in phototransduction [17]. When Lewis rats were immunized with recoverin, it induced uveoretinitis [13], which is not a feature of AIR where patients do not have cell or flare clinically. When anti-recoverin antibody was injected intravitreally into *anesthetized* monkey, rat, and rabibit eyes, the antibody caused retinal cell death by apoptosis in vivo [18] and in vitro [19,20].

To better understand the role of anti-recoverin antibodies in AIR, we first tried immunizing C57BL/6J mice with recoverin, but found minimal expression of anti-recoverin antibody and no ERG changes. We chose B6.MRL-*Fas^lpr^*/J mice (LPR) for immunization with recoverin because upon screening twenty mouse strains for anti-retinal antibodies it was the only strain in which we found naturally occurring (faint) anti-retinal antibodies (data not shown). B6.MRL-Fas^lpr^/J mice have a lymphoproliferation spontaneous mutation (*Fas^lpr^*) that show systemic autoimmunity and are regarded as a mouse model for lupus [21]. We chose this strain to investigate if an AIR model could be induced by immunization with recoverin. A second approach in our search for murine models of AIR was to create recoverin-sensitized hybridoma cells and then to implant the hybridoma cells in balb/cJ mice. This approach allowed us to examine if high levels of circulating anti-recoverin antibody would produce a retinopathy.

## Methods

### Animals

All studies involving mice were approved by the University Committee on the Use and Care of Animals at the University of Michigan. B6.MRL-*Fas^lpr^*/J (LPR) mice, C57BL/6J mice, and balb/cJ mice were obtained from Jackson Laboratory, Bar Harbor, ME. Sample sizes of six for each group were chosen due to the scarcity of recoverin protein for immunization.

### Purification of recombinant recoverin

A mouse recoverin-expressing construct was made in pET11a-Rec (kindly provided by Dr. James Hurley). Recombinant recoverin was purified as described previously [22]. Briefly, a recoverin construct was transformed and expressed in *E. coli* strain BL21 (DE3) pLysS (Novagen, Merck, Darmstadt, Germany). The culture was grown in 250 ml of LB (Luria–Bertani) medium at 37 °C containing ampicillin (50 μg/ml). Protein expression was induced with 1 mM isopropyl α-d-thiogalactopyranoside and cells were incubated for an additional 3 h at room temperature. Cells were then harvested by centrifugation and re-suspended in 10 ml of buffer A (50 mM Hepes (pH 7.5), 100 mM NaCl, 1 mM CaCl_2_, 5 mM α-mercaptoethanol, and 0.1 mM PMSF). After sonication and centrifugation (20,000× g for 30 min at 4 °C), the cleared lysate was loaded onto a buffer A-preequilibrated Econ-column (10 mm id×10 cm; Bio-Rad, Hercules, CA) with 5 ml of Phenyl Sepharose 6 Fast Flow (low sub resin; Amersham Biosciences, GE healthcare, Piscataway, NJ) at a flow rate of 0.4 m/min. The column was washed (2 ml/min) with at least 10 column volumes of buffer A to remove nonspecifically adsorbed proteins. Fractions of purified recoverin were eluted (0.4 ml/min) with buffer B (5 mM Hepes pH 7.5, 100 mM NaCl, 5 mM α-mercaptoethanol, and 5 mM EGTA) and stored at −70 °C until use.

### Hybridoma cell generation

Six-week-old balb/cJ mice were used for hybridoma production. In brief, 50 μg recoverin protein in 200 µl of PBS or 200 µl of PBS (for control mice) were mixed with equal volumes of Complete Freund's adjuvant (Pierce, Rockford, IL). The mixed emulsion was injected intraperitoneally on day 0; a second dose of 50 µg recoverin protein in an emulsion with Incomplete Freund's adjuvant (Pierce) was injected intraperitoneally on days 14 and 42. When the serum antibody titer reached twice the background value at 1:10,000 dilution, single spleen cells from the immunized mice were fused with previously prepared myeloma cells [23]. Fusion is accomplished by co-centrifuging freshly harvested spleen and myeloma cells in the presence of polyethylene glycol. The cells were then distributed to 96-well plates containing feeder cells. The newly formed small clusters of hybridoma cells from the 96-well plates were then grown in tissue culture flasks and clone screening was performed to determine which cultures were producing recoverin antibodies.

### Mouse model

Six-week-old *LPR* mice or C57BL/6J mice were injected intraperitoneally with 50 µg recoverin (n=6) or PBS (n=6; control mice) in an emulsion with Complete Freund's adjuvant containing Mycobacterium tuberculosis (1:1, v/v; Pierce) on day 0; 50 µg protein in an emulsion with Incomplete Freund's adjuvant (1:1, v/v; Pierce) were injected intraperitoneally on days 14 and 42. On day 49, tail blood was taken using capillary tubes (Sarstedt, Numbrecht, Germany), ERGs were recorded, and the mouse eyes were enucleated after euthanasia (ketamine [80 mg/kg] and xylazine [16 mg/kg]) for histopathologic examination and flow cytometric analysis.

### Hybridoma model

Five-month-old balb/cJ mice were given pristane (500 μl) two weeks before the intraperitoneal injection of hybridoma cells (5×10^6^ cells; n=6). Ascites formed 7–10 days after the injection and were withdrawn for antibody testing. Next, ERGs were recorded and the mice eyes were taken as above for histology.

### Electroretinography

Mice were dark-adapted overnight and anesthetized with an intraperitoneal injection of normal saline solution containing ketamine (80 mg/kg) and xylazine (16 mg/kg). Next, ERGs were recorded from the corneal surface after pupil dilation (0.1% atropine and 0.1% phenylephrine HCl) using a gold loop electrode referenced to a gold wire in the mouth. An electrode placed in the tail served as a ground. A drop of methylcellulose (2.5%) was placed on the corneal surface to ensure electrical contact and to maintain corneal integrity. Body temperature was maintained at a constant temperature of 38 °C using a heated water pad. All stimuli were presented in a Ganzfeld dome (LKC Technologies, Gaithersburg, MD). Rod-dominated responses to white flashes of light over a 4.0–5.0 log unit range of intensities were recorded. Cone-dominated responses were obtained with white flashes over a 2.0 log unit range of intensities at 2.1 Hz on a rod-saturating background (1.46 log cd/m^2^) after 10 min of exposure to the background light to allow for complete light adaptation. Responses were amplified and filtered (0.03–10,000 Hz) and digitized using an I/O board (model PCI-1200; National Instruments, Austin, TX) in a personal computer. Evoked responses were sampled every 0.5 ms over a response window of 240 ms. For each stimulus condition, responses were computer-averaged with up to 20 records averaged for the weakest signals.

### Immunoblot analysis

Purified recoverin (0.2 µg) was applied to 4%–20% linear gradient Tris–HCl gel (Bio-Rad) and transferred onto nitrocellulose membranes (Bio-Rrad). The membranes were blocked with 5% milk and incubated with samples of analyzed sera diluted 1:1,000. Immune complexes were detected with anti-mouse IgG goat horseradish peroxidase-conjugated secondary antibodies (SouthernBiotech, Birmingham, AL) and enhanced with chemiluminescent substrate (Pierce).

### ELISA for serum antibodies to recoverin

Serum antibodies to recoverin were quantified by enzyme-linked immunosorbent assay (ELISA). Briefly, microtiter plates were coated with 100 ng/well of recoverin in 0.1 M Tris-HCl buffer, pH 9.0, overnight at room temperature. Following five washes with phosphate buffer solution (PBS), the plates were coated with 1% skim milk in PBS for 2 h at room temperature and then were incubated with 50 μl of diluted serum in PBS for 2 h at room temperature. After five washes with PBS containing 0.05% Tween-20, the plates were incubated with 50 µl of goat anti-human IgG antibody conjugated with horseradish peroxidase (SouthernBiotech) at room temperature for 2 h. Following five washes with PBS containing 0.05% Tween-20, bound reactants were developed with 50 µl of 50 µg/ml 2,2'-azinobis diammonium salt (Pierce) in citrate-phosphate buffer (pH 4.0; 0.2M Na_2_HPO_4_, 0.1M citrate, 1.0 ìl/10 ml of 30% H_2_O_2_). The absorbance was determined at 405 nm.

### Light microscopy

Eyes were immediately removed and immersed in a cold fixative of 4% glutaraldehyde in 0.1 M phosphate buffer. Corneas were removed and the eyes left in fixative for 24 h. The lens was then removed, followed by dehydration with a graded series of increasing ethanol concentrations. Eyecups were embedded in an Epon mixture. For each sample, 0.5 µm-thin sections were stained with toluidine blue for light microscopy.

### Immunohistochemistry

Frozen sections were obtained from retina embedded in Optimal Cutting Temperature (OCT; Tissue-Tek; Miles Inc., Elkhart, IN). Sections were blocked for non-specific protein-binding with 3% BSA in PBS at room temperature for 1 h and then incubated with the first antibody for 2 h at room temperature in PBS containing 3% BSA. After washing with PBS+0.2% Triton X-100 (room temperature, 3×10 min), the sections were incubated with the fluorescence-conjugated secondary antibody in PBS+3% BSA. They were then counterstained with 4',6-diamidino-2-phenylindole (DAPI; 0.3 µM) before observation with a fluorescence microscope (Olympus, Tokyo, Japan). The anti-glial fibrillary acidic protein (GFAP) antibody (1:500 dilution) was from Abcam (Cambridge, MA), the anti-rhodopsin (1:500 dilution), anti-S-opsin, and anti-calretinin antibodies (1:500 dilution) were from Millipore (Billerica, MA), the anti PKC-α antibody was from Sigma (St. Louis, MO), and the anti-C1q and anti-C3 antibodies (1:500 dilution) were from Cederlane (Burlington, NC).

### Flow cytometry

White blood cells and macrophages were obtained from control and AIR mice retinas by vigorous pipetting and were centrifuged. Staining for flow cytometry was performed within 24 h. Briefly, fluorochrome-conjugated mAbs were added to cells (1 µg/10^6^ cells). These were then incubated in the dark for 20 min at room temperature. Cells were washed twice with staining buffer, resuspended in Cytofix (BD Biosciences, San Jose, CA), and kept in the dark at 4 °C until cytometric analysis (within 24 h). Analysis was performed on a FACSCalibur flow cytometer (BD Biosciences). Mean fluorescent intensity (MFI) was calculated as a ratio of mean fluorescence sample/isotype fluorescence. Percentage positive expression was calculated by comparing the population of cells with increased fluorescent intensity to the following isotypes: anti-CD4, -CD8, -CD19, -CD69; anti-Ly6G antibodies were from BD Biosciences and the anti-mouse CD68 antibody (1:500 dilution) was from Fisher Scientific (Pittsburgh, PA).

### Statistical analysis

The student *t*-test was employed for the statistical analysis. Statistical significance was defined as p<0.05. All data were presented as mean±SD.

## Results

### Purification of recombinant recoverin and generation of recoverin hybridoma cells

**Figure 1:**
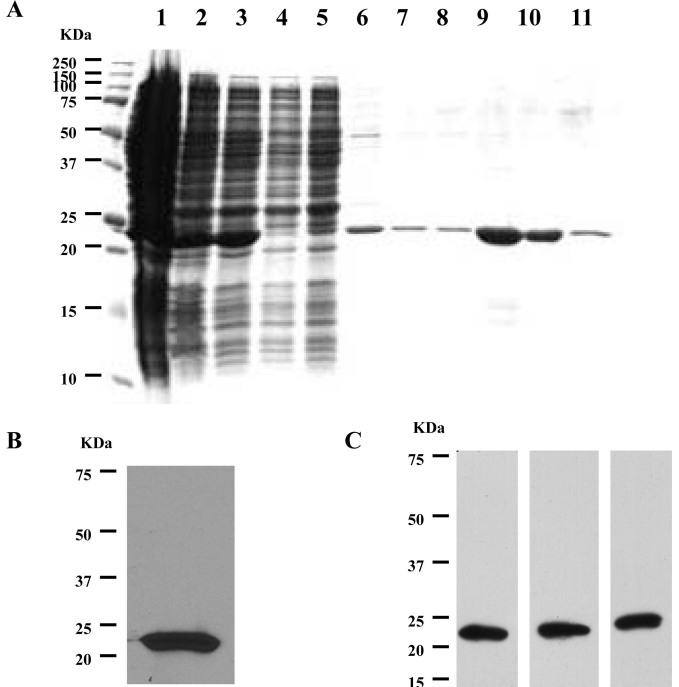
Purification of recombinant recoverin and generation of recoverin hybridoma cells. **A**: Sodium dodecyl sulfate PAGE (SDS–PAGE) analysis of the fractions during the recoverin purification process. 1) crude extract; 2) after centrifugation; 3) after filtration; 4 and 5) apply to column; 6) wash column; 7–11) elution. **B**: Immunoblot analysis of the eluted fractions of recoverin. **C**: Immunoblot analysis of the ascites from three balb/cJ mice injected with recoverin hybridoma cells. Molecular size standards are shown on the left.

We first purified the recombinant recoverin using a Phenyl Sepharose 6 Fast Flow column. The purity of recoverin was confirmed by SDS–PAGE analyses of the supernatant during the recoverin purification process (Figure 1A) and the purified recombinant recoverin was confirmed by immunoblotting (Figure 1B). We also generated recoverin monoclonal antibody-producing hybridoma cells and then injected the cells intraperitoneally in five-month old balb/cJ mice. Ascites formed after about 10 days [23] and the ascitic fluid was withdrawn to test the recoverin antibody level (Figure 1C).

### Recoverin-associated retinopathy mouse models

**Figure 2:**
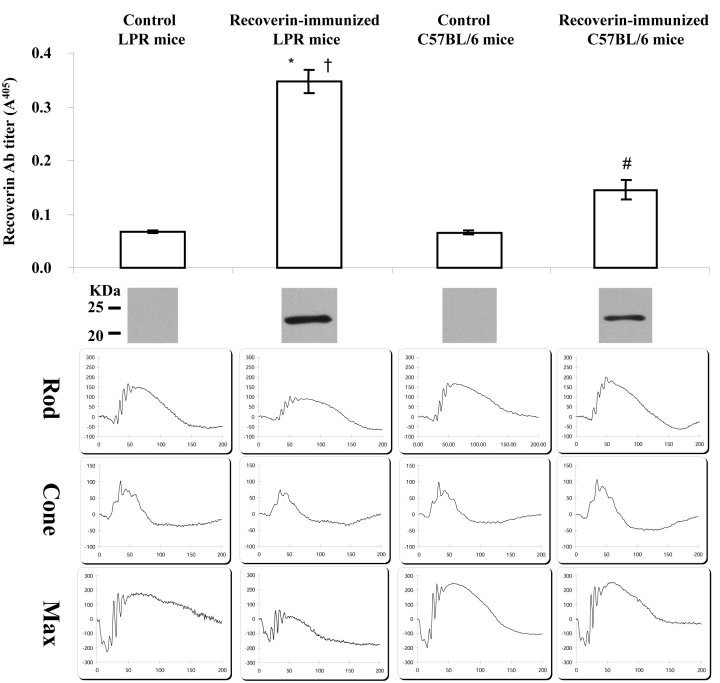
Accelerated anti-recoverin antibodies in recoverin-immunized B6.MRL-Fas*^lpr^*/J (*LPR*) mice associated with attenuated electroretinogram (ERG) responses. Although there was an increase of anti-recoverin antibodies in recoverin-immunized C57BL/6J mice, the ERG responses were still normal compared with control C57BL/6J mice. The antibody level was significantly enhanced in recoverin-immunized *LPR* mice compared with recoverin-immunized C57BL/6J mice, accompanied by attenuated scotopic and photopic ERG responses. A serum titer (1:10,000 dilution) of anti-recoverin antibodies was analyzed by enzyme-linked immunosorbent assay (ELISA; upper) and western blot (middle); ERG responses were also analyzed (bottom). There were significant differences in antibody levels by ELISA in recoverin-immunized *LPR* mice versus control *LPR* mice (p<0.001); recoverin-immunized *LPR* mice versus recoverin-immunized C57BL/6J mice (p<0.001); recoverin-immunized C57BL/6J mice versus control C57BL/6J mice (p<0.05).

**Figure 3:**
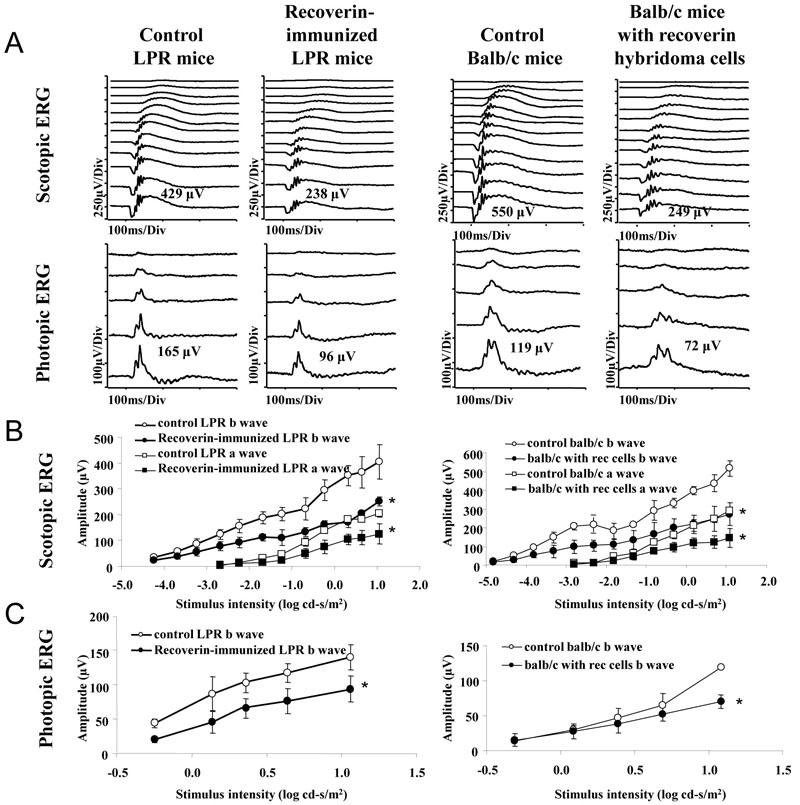
Attenuated electroretinogram (ERG) response in recoverin-immunized B6.MRL-*Fal^lpr^*/J mice (LPR) mice and balb/cJ mice injected with recoverin hybridoma cells. Reduced ERG responses from recoverin-immunized *LPR* mice and balb/cJ mice injected with recoverin hybridoma cells was observed under dark-(scotopic ERG; **A**, **B**) and light-adapted (photopic ERG; **A**, **C**) conditions. Intensity-response curves of the amplitude of flash b-wave, a-wave from the dark-adapted ERG was shown in **B**, and b-wave amplitudes from the photopic ERG are depicted in **C**. The x-axes indicate stimulus intensity (log cd-s/m^2^). The y-axes indicate amplitude (µV). Student *t*-test showed multiple significant differences between experimental groups compared to control values (see Table 1).

To test whether pathologic retinopathy could be generated by immunizing recoverin in-C57BL/6J mice and B6.MRL-*Fas^lpr^*/J (*LPR*) mice, we initially challenged six-week-old mice with 50 µg recoverin intraperitoneally and repeated this two more times over eight weeks, at which time the antibody levels were tested. Anti-recoverin antibody levels were significantly increased in recoverin-immunized *LPR* mice compared to control *LPR* mice (p<0.05), while the antibody titer was ~2 times higher in recoverin-sensitized *LPR* mice compared to recoverin-sensitized C57BL/6J mice (Figure 2). Similar results were found in the immunoblotting analysis (Figure 2). These data indicate higher anti-recoverin antibody levels can be achieved in *LPR* mice than in C57BL/6J mice using the same methodology. Electroretinogram recordings were used to determine whether immunization with recoverin affects the retinal function in C57BL/6J and *LPR* mice. However, small but significant electroretinographic changes (p<0.05) in recoverin-immunized C57BL/6J mice were observed in the scotopic, photopic, and maximum responses compared to control C57BL/6J mice (Figure 2). However, degeneration was not detected upon histology and the ERG changes in the immunized C57BL/6J mice revealed a small physiologic effect of the autoantibodies. By comparison, the histologic changes in the LPR mice suggest that higher elevations of anti-recoverin antibodies are needed to elicit disease. However, the ERG amplitudes of recoverin-immunized mice for the scotopic, photopic, and maximum electroretinographic responses were significantly reduced by ~35% compared with control LPR mice (Figure 2, Figure 3; inset table in Figure 3).

**Table 1:** Statistical analysis of electroretinogram (ERG) responses in recoverin-immunized B6.MRL-*Fal^lpr^*/J (LPR) mice and balb/c mice injected with recoverin hybridoma cells compared to control mice values.

**ERG Parameters**	**Recoverin-immunized LPR mice versus control LPR mice**	**Balb/c anti-recoverin hybridoma mice versus control balb/c mice**
Rod-isolated b-wave	p<0.007	p<0.001
Dark Max a-wave	p<0.002	p<0.024
Dark Max b-wave	p<0.003	p<0.001
Photopic b-wave	p<0.002	p<0.001

n=6 for each group.

We also injected the recoverin hybridoma cells intraperitoneally in five-month old balb/cJ mice. Ascites formed after about 10 days and the ascitic fluid was withdrawn to test the recoverin antibody level (Figure 1C); the ERG was also tested. Both b- and a-wave amplitudes in scotopic electroretinographic responses were reduced by about 50% in mice who received sham intraperitoneal injections compared to control balb/cJ mice; b-wave amplitudes in photopic electroretinographic responses were reduced by ~40% (Figure 3).

### Retinal changes in recoverin-associated retinopathy mouse models

**Figure 4:**
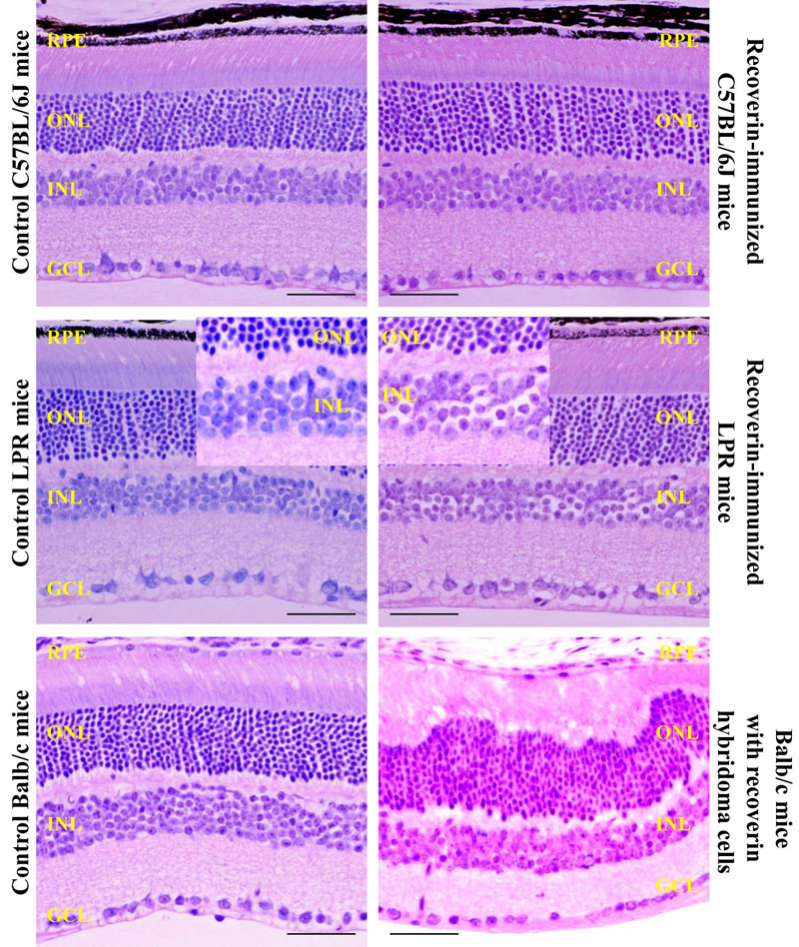
Histology changes. Retinal histology showed swollen cell bodies in the inner nuclear layer in recoverin-immunized B6.MRL-*Fal^lpr^*/J (*LPR)* mice, greater than the mild changes observed in the controls. Photoreceptor and outer nuclear layer waves were observed in the retina of balb/cJ mice injected with recoverin hybridoma cells. Migrating cells between the inner nuclear layer (INL) and ganglion cell layer (GCL) were also observed in these two mouse models. No change was observed in recoverin-immunized C57BL/6J mice. Abbreviations: ONL is the outer nuclear layer; RPE is retinal pigment epithelium. Scale bar equal to 50 µm.

After LPR mice were sensitized with recoverin three times, we evaluated the mouse eyes using biomicroscopy (25×) and indirect retinoscopy; there were no cells in the aqueous or vitreous, which excluded active uveitis. The eyes were then enucleated for histopathologic analysis two weeks after the last immunization. In the recoverin-sensitized LPR mice, H&E staining showed cell body swelling throughout the inner nuclear layer (INL) and migrating cells between the INL and ganglion cell layer (GCL) were also observed (Figure 4). In LPR-sensitized mice, GFAP staining demonstrated

**Figure 5:**
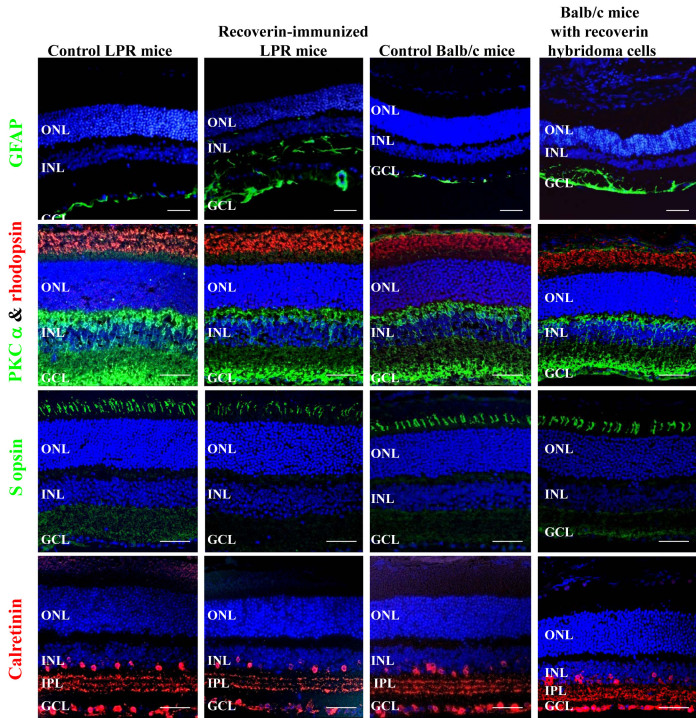
Immunohistogic identification of retinal changes in recoverin-immunized B6.MRL-*Fal^lpr^*/J (*LPR)* mice and balb/cJ mice injected with recoverin hybridoma cells. Glial fibrillary acidic protein (GFAP) staining detected a marked increase of Muller cell reactive gliosis in recoverin-immunized *LPR* mice and balb/cJ mice injected with recoverin hybridoma cells, which indicated retinal degeneration. Rhodopsin and S-opsin expression were not altered in these groups, while bipolar cells (protein kinase alpha [PKC-α]) were decreased in both test groups. There is no change in amacrine cells (calretinin) in these two mouse models, however the inner plexiform layer were reduced. Cell nuclei were stained with 4',6-diamidino-2-phenylindole (DAPI; blue). Abbreviations: GCL represents ganglion cell layer; IPL represents inner plexiform layer; INL represents Inner nuclear layer; ONL represents outer nuclear layer; RPE represents retinal pigment epithelium. Scale bar equal to 50 µm.

Mueller and astrocyte swelling and increased numbers in the INL and GCL compared to normal controls (Figure 5; GFAP). Rhodopsin and S-opsin expression were not altered in either group, while bipolar cells (stained with protein kinase C-α antibodies) were decreased in recoverin-immunized *LPR* mice (Figure 5). Although amacrine cells showed no change, the interplexiform layer (IPL) was thinned compared to controls.

H&E staining from hybridoma-injected balb/cJ mice showed photoreceptor and outer nuclear layer (ONL) thickening that was uneven in distribution (Figure 4), as well as migrating cells between the INL and GCL. Glial fibrillary acidic protein staining showed a remarkable increase in astrocyte and glial staining from the INL to the GCL (Figure 5). Rhodopsin and S-opsin expression were not altered in either group, while bipolar cells (PKC-α) were decreased in the hybridoma-injected balb/cJ mice. Amacrine cells were not changed, but the IPL was noticeably thinned. (Figure 5)

The complement system is known to participate in a variety of ocular diseases, including macular degeneration and uveitis [24,25]. Complement components contribute to pathogenic processes by damaging the tissue and stimulating chemotaxis and in age-related macular degeneration (AMD) are known to facilitate neovascularization. C1q is able to directly bind to apoptotic cells via its globular head domains, which may induce complement activation with subsequent opsonization of C4b and C3b to the surface of apoptotic cells [26]. To test if complement components were involved in the mechanism of our two recoverin-associated AIR mouse models, we stained for C1q and C3.

**Figure 6:**
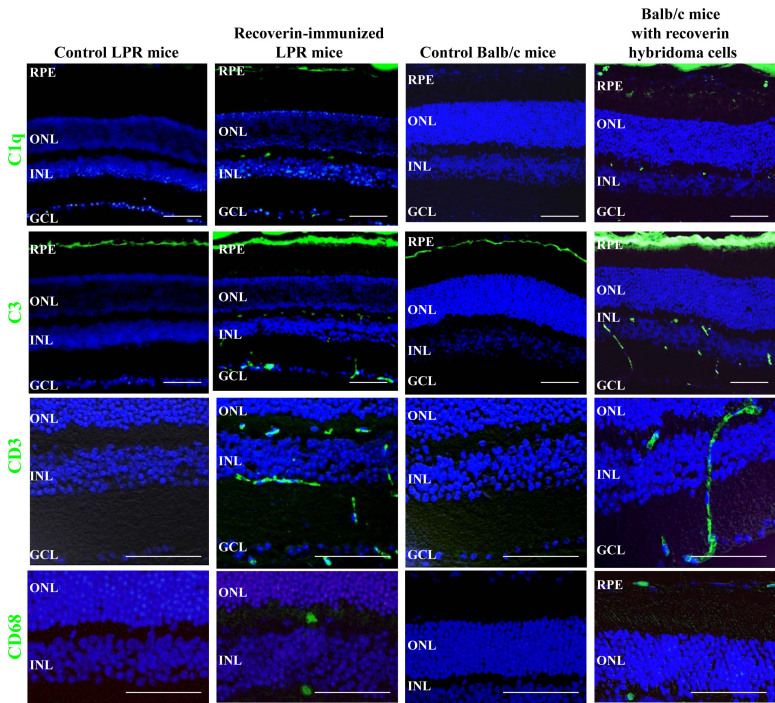
Inflammatory changes in recoverin-immunized B6.MRL-*Fal^lpr^*/J (*LPR)* mice and hybridoma balb/cJ mice. Increase of C1q and C3 deposition and infiltration of CD3+ and CD68+ cells in the retina of recoverin-immunized *LPR* mice and *Balb*/c mice injected with recoverin hybridoma cells. No C1q deposition and C3 deposition in only retinal pigment epithelium (RPE) was observed in control LPR mice, while they were enhanced in the RPE, inner nuclear layer (INL), and ganglion cell layer (GCL) of recoverin-immunized LPR mice retina. No C1q or C3 deposition in RPE was observed in control *Balb*/c mice, while some C1q deposition was observed in the RPE, INL and GCL of the retina of *Balb*/c mice injected with recoverin hybridoma cells, and a marked increase of C3 deposit on RPE and INL was observed. CD3+ cells were observed between the INL and GCL layers, and the migrating cells between INL and GCL were CD3+ cells. While CD68+ cells were found in inner plexiform layer (IPL) and outer plexiform layer (OPL) in recoverin-immunized LPR mice retina, they were found in RPE and OPL in *Balb*/c mice injected with recoverin hybridoma cells. Cell nuclei were stained with 4',6-diamidino-2-phenylindole (DAPI; blue). Abbreviations: ONL, outer nuclear layer. Scale equal to 50 µm.

Immunohistochemical analyses showed that compared with control *LPR* mice, scattered C1q deposits were observed in the retinal pigment epithelium (RPE), INL, and GCL of recoverin-immunized *LPR* mice (Figure 6). No sections of the control *LPR* mice exhibited C1q deposits in the retina (Figure 6). Although there were C3 deposits in the RPE of control *LPR* mice (Figure 6), C3 deposits were remarkably increased in the RPE layer in recoverin-immunized *LPR* mice and scattered C3 deposits were found in the INL and GCL (Figure 6). CD3+ cells were observed between the INL and GCL layers, and the migrating cells between INL and GCL were CD3+ cells (Figure 6). CD68+ cells were found in the IPL and OPL in recoverin-immunized *LPR* mice retina (Figure 6).

Immunohistologic microscopy showed that compared with control balb/cJ mice, very few scattered C1q deposits were found in the RPE, INL, and GCL of recoverin monoclonal antibody-producing hybridoma cells injected in the balb/cJ mice (Figure 6). There was no C1q immunostaining in control balb/cJ mice. The staining for C3 deposits was remarkably increased in the RPE layer in recoverin monoclonal antibody producing hybridoma cells injected into balb/cJ mice compared to control mice; scattered C3 deposits in the INL and GCL were also seen. (Figure 6). The migrating cells between the INL and GCL were CD3+ cells (Figure 6), and CD68+ cells were found in the RPE and OPL of the retinas of *Balb/c* mice injected with recoverin hybridoma cells (Figure 6).

**Figure 7:**
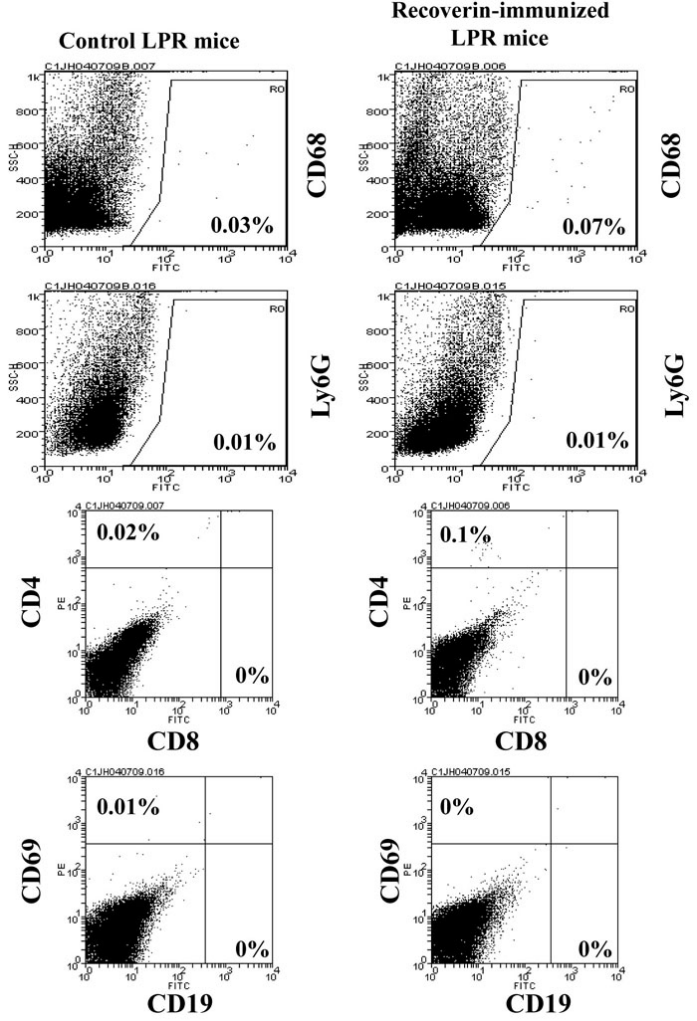
Retinal infiltrate-cell analysis by flow cytometry. CD68 and CD3 positive cells were increased in recoverin-immunized LPR mice compared with control B6.MRL-*Fal^lpr^*/J (LPR), While no Ly6G positive cells and CD19 positive cells were found in both.

Flow cytometry analysis of CD 68 (macrophage marker) showed increased levels of CD68 positive cells (0.07%) in the retinas of recoverin-immunized *LPR* mice compared to control *LPR* mice (0.03%) (Figure 7). CD3 positive cells increased (0.1%) in the retinas of recoverin-immunized *LPR* mice compared to those of control *LPR* mice (0.02%). There were no Ly6G positive cells, CD8 positive cells, or CD19 positive cells observed in either group.

## Discussion

Although AIR has been identified for over 15 years, an animal model of this disease has not been available. Animal models, and particularly mice with identical or very similar hereditary disorders to man, have proven to be a powerful investigative tool for understanding cellular, molecular, and degenerative mechanisms in retinal degeneration. With these two AIR models it will be possible to provide new information about the pathophysiology of AIR, specifically looking at inflammatory mechanisms in the retina and testing different putative pathologic retinal antigens known to be associated with AIR. In this study, we described two mouse models with immunologically induced retinopathy associated with elevated recoverin antibodies; both models had attenuated scotopic and photopic ERGs, retinal thinning, accelerated C1q and C3 deposits in the retinas, enhanced expression of GFAP staining identifying a reactive gliosis, and infiltration of inflammatory cells, including macrophages and neutrophils.

Sixty-five percent of patients with AIR have a family history of other autoimmune disorders (e.g., rheumatoid arthritis, lupus, thyroid disease, asthma) and having an autoimmune genetic background seems to make some patients more susceptible to AIR [9]. The recoverin-immunized *LPR* mice showed moderate retinal degeneration that was not found in the recoverin-immunized C57BL/6J mice (with no autoimmune genetic background), similar to the pattern seen in human AIR. Here, we only tested one retinal antigen to stimulate antibody formation, while AIR patients normally have multiple immunoreactive bands from circulating anti-retinal antibodies on western blot testing. Identification of the underlying antigenic proteins in AIR has been slow, and only a few of the many antibodies found in AIR patients have been proven to cause retinal damage or degeneration. Most immunoreactive bands (i.e., antibodies) from AIR patients have not been investigated and their antigenic stimuli have yet to be identified.

Recoverin has been proven to be a cause of AIR [3,10,12,13]. A rat model system has been established to elucidate the contribution of recoverin autoantibodies to the development of retinopathy [13]. The injection of recoverin has led to the induction of uveitis and retinal degeneration. In a guinea pig model, animals were sensitized with small cell lung cancer cell lines that induced production of anti-recoverin autoantibodies in the animals and caused retinopathy [27]. In an experimental BALB/c mouse model, Maeda et al. induced specific cytotoxic T lymphocytes in response to the peptide R64 (AYQHVFRSF) and observed a tumor-preventive effect; however, this peptide also induced the generation of antibodies and retinal degeneration [28]. Our two mouse models have elevated circulating anti-recoverin antibodies, which is a feature observed in AIR patients, especially CAR patients. In other studies, anti-recoverin autoantibodies induced apoptosis in photoreceptor and bipolar cells, leading to retinal degeneration [29,30]. We did not observe apoptotic cells in these two models (data not shown), but we only looked at one time point after the disease was clearly present on the ERG. Serial Terminal deoxynucleotidyl transferase dUTP nick end labeling (TUNEL) and autophagy studies will be needed in the future to further investigate this issue. It is unclear why the blood-retinal barrier does not prevent autoantibodies from penetrating into the retina, but leukostasis or leukocyte deposits (focal vascular staining [31]) on retinal vessel walls seen in AIR patients may damage the tight junctions and allow immunoglobulin diffusion [31,32].

One of the more important findings of this study was the striking reactive retinal gliosis and infiltration by leukocytes found in both models. Differential changes in the GFAP expression in Mueller glial cells is the most sensitive nonspecific response to retinal diseases and injuries and has been used as a universal early cellular marker for retinal injury [33]. The finding of inflammatory cells on retinal histopathology and flow cytometry in our two mouse models is consistent with recent case reports of AIR in systemic lupus erythematosus. Macrophages were seen in the retina, particularly in areas of photoreceptor degeneration [34]. Activation of infiltrating macrophages and granulocytes causes destruction of the retina by the release of reactive oxygen species, including nitric oxide (NO) and superoxide [35]. Humans with AIR do not have cells or flare in the anterior chamber and seldom show obvious clinical infiltration of the retina, although cystoid edema and retinal wrinkling are commonly found [4]. White retinal patches are seldom a feature in AIR. The new finding of a gliotic cellular response and infiltration of retinas by inflammatory cells without uveitis is important and is likely to bear on the pathologic degenerative mechanisms seen in AIR.

The complement system is involved in antigen-specific immune responses, with an identified role in antigen processing and presentation, T-cell proliferation and differentiation, and B-cell activation [36]. Complement activation products such as C3 have been shown to be present in the eyes of patients with autoimmune uveitis [24] and in drusen in patients with age-related macular degeneration [25]. Our two mouse models show elevated C1q and C3 deposition in the RPE and INL of retinas. The complement components may enhance the damage initiated by the pathogenic process. These preliminary results suggest further investigation is needed to determine the role of the complement system in AIR.

### Conclusion

Two successful mouse models for AIR were generated by our experimental approaches, both using alternate methods of producing anti-recoverin antibodies. These models not only will be important for understanding the pathogenesis and mechanisms involved in human AIR, but will also be valuable for testing potential therapies. The pathologic changes in anti-recoverin antibodies were not as severe as we had expected, which may suggest that a mixture of retinal antibodies may be the cause of the varying clinical severity seen in AIR patients who typically have multiple ARAs.

## References

[1] Heckenlively JR Ferreyra HA Autoimmune retinopathy: a review and summary. Semin Immunopathol 2008 30 127 34 18408929 10.1007/s00281-008-0114-7

[2] Keltner JL Thirkill CE Yip PT Clinical and immunologic characteristics of melanoma-associated retinopathy syndrome: eleven new cases and a review of 51 previously published cases. J Neuroophthalmol 2001 21 173 87 11725182 10.1097/00041327-200109000-00004

[3] Thirkill CE Tait RC Tyler NK Roth AM Keltner JL The cancer-associated retinopathy antigen is a recoverin-like protein. Invest Ophthalmol Vis Sci 1992 33 2768 72 1388144

[4] Heckenlively JR Jordan BL Aptsiauri N Association of antiretinal antibodies and cystoid macular edema in patients with retinitis pigmentosa. Am J Ophthalmol 1999 127 565 73 10334350 10.1016/s0002-9394(98)00446-2

[5] LeHoang P Cassoux N George F Kullmann N Kazatchkine MD Intravenous immunoglobulin (IVIg) for the treatment of birdshot retinochoroidopathy. Ocul Immunol Inflamm 2000 8 49 57 10806434

[6] Audo I Robson AG Holder GE Moore AT The negative ERG: clinical phenotypes and disease mechanisms of inner retinal dysfunction. Surv Ophthalmol 2008 53 16 40 18191655 10.1016/j.survophthal.2007.10.010

[7] Schuster A Apfelstedt-Sylla E Pusch CM Zrenner E Thirkill CE Autoimmune retinopathy with RPE hypersensitivity and 'negative ERG' in X-linked hyper-IgM syndrome. Ocul Immunol Inflamm 2005 13 235 43 16019685 10.1080/09273940590928571

[8] Suhler EB Chan CC Caruso RC Schrump DS Thirkill C Smith JA Nussenblatt RB Buggage RR Presumed teratoma-associated paraneoplastic retinopathy. Arch Ophthalmol 2003 121 133 7 12523906 10.1001/archopht.121.1.133

[9] Ferreyra HA Jayasundera T Khan NW He S Lu Y Heckenlively JR Management of autoimmune retinopathies with immunosuppression. Arch Ophthalmol 2009 127 390 7 19365013 10.1001/archophthalmol.2009.24

[10] Adamus G Autoantibody targets and their cancer relationship in the pathogenicity of paraneoplastic retinopathy. Autoimmun Rev 2009 8 410 4 19168157 10.1016/j.autrev.2009.01.002 PMC2680817

[11] Lu Y Jia L He S Hurley MC Leys MJ Jayasundera T Heckenlively JR Melanoma-associated retinopathy: a paraneoplastic autoimmune complication. Arch Ophthalmol 2009 127 1572 80 20008709 10.1001/archophthalmol.2009.311 PMC4618318

[12] Heckenlively JR Fawzi AA Oversier J Jordan BL Aptsiauri N Autoimmune retinopathy: patients with antirecoverin immunoreactivity and panretinal degeneration. Arch Ophthalmol 2000 118 1525 33 11074809 10.1001/archopht.118.11.1525

[13] Adamus G Ortega H Witkowska D Polans A Recoverin: a potent uveitogen for the induction of photoreceptor degeneration in Lewis rats. Exp Eye Res 1994 59 447 55 7859820 10.1006/exer.1994.1130

[14] Adamus G Brown L Weleber RG Molecular biomarkers for autoimmune retinopathies: significance of anti-transducin-alpha autoantibodies. Exp Mol Pathol 2009 87 195 203 19744478 10.1016/j.yexmp.2009.08.003 PMC2783245

[15] Ohguro H Yokoi Y Ohguro I Mamiya K Ishikawa F Yamazaki H Metoki T Takano Y Ito T Nakazawa M Clinical and immunologic aspects of cancer-associated retinopathy. Am J Ophthalmol 2004 137 1117 9 15183799 10.1016/j.ajo.2004.01.010

[16] Weleber RG Watzke RC Shults WT Trzupek KM Heckenlively JR Egan RA Adamus G Clinical and electrophysiologic characterization of paraneoplastic and autoimmune retinopathies associated with antienolase antibodies. Am J Ophthalmol 2005 139 780 94 15860281 10.1016/j.ajo.2004.12.104

[17] Kawamura S Rhodopsin phosphorylation as a mechanism of cyclic GMP phosphodiesterase regulation by S-modulin. Nature 1993 362 855 7 8386803 10.1038/362855a0

[18] Ohguro H Ogawa K Maeda T Maeda A Maruyama I Cancer-associated retinopathy induced by both anti-recoverin and anti-hsc70 antibodies in vivo. Invest Ophthalmol Vis Sci 1999 40 3160 7 10586938

[19] Adamus G Machnicki M Seigel GM Apoptotic retinal cell death induced by antirecoverin autoantibodies of cancer-associated retinopathy. Invest Ophthalmol Vis Sci 1997 38 283 91 9040460

[20] Lei B Bush RA Milam AH Sieving PA Human melanoma-associated retinopathy (MAR) antibodies alter the retinal ON-response of the monkey ERG in vivo. Invest Ophthalmol Vis Sci 2000 41 262 6 10634629

[21] Morse HC 3rd Roths JB Davidson WF Langdon WY Fredrickson TN Hartley JW Abnormalities induced by the mutant gene, lpr. Patterns of disease and expression of murine leukemia viruses in SJL/J mice homozygous and heterozygous for lpr. J Exp Med 1985 161 602 16 2982991 10.1084/jem.161.3.602 PMC2187576

[22] Dizhoor AM Ericsson LH Johnson RS Kumar S Olshevskaya E Zozulya S Neubert TA Stryer L Hurley JB Walsh KA The NH2 terminus of retinal recoverin is acylated by a small family of fatty acids. J Biol Chem 1992 267 16033 6 1386601

[23] Kearney JF Radbruch A Liesegang B Rajewsky K A new mouse myeloma cell line that has lost immunoglobulin expression but permits the construction of antibody-secreting hybrid cell lines. J Immunol 1979 123 1548 50 113458

[24] Mondino BJ Glovsky MM Ghekiere L Activated complement in inflamed aqueous humor. Invest Ophthalmol Vis Sci 1984 25 871 3 6610667

[25] Nozaki M Raisler BJ Sakurai E Sarma JV Barnum SR Lambris JD Chen Y Zhang K Ambati BK Baffi JZ Ambati J Drusen complement components C3a and C5a promote choroidal neovascularization. Proc Natl Acad Sci USA 2006 103 2328 33 16452172 10.1073/pnas.0408835103 PMC1413680

[26] Nauta AJ Trouw LA Daha MR Tijsma O Nieuwland R Schwaeble WJ Gingras AR Mantovani A Hack EC Roos A Direct binding of C1q to apoptotic cells and cell blebs induces complement activation. Eur J Immunol 2002 32 1726 36 12115656 10.1002/1521-4141(200206)32:6<1726::AID-IMMU1726>3.0.CO;2-R

[27] Thirkill CE Experimental, cancer-induced retinopathy. Ocul Immunol Inflamm 1997 5 55 65 9145694 10.3109/09273949709085051

[28] Maeda A Maeda T Ohguro H Palczewski K Sato N Vaccination with recoverin, a cancer-associated retinopathy antigen, induces autoimmune retinal dysfunction and tumor cell regression in mice. Eur J Immunol 2002 32 2300 7 12209643 10.1002/1521-4141(200208)32:8<2300::AID-IMMU2300>3.0.CO;2-7

[29] Adamus G Machnicki M Elerding H Sugden B Blocker YS Fox DA Antibodies to recoverin induce apoptosis of photoreceptor and bipolar cells in vivo. J Autoimmun 1998 11 523 33 9802939 10.1006/jaut.1998.0221

[30] Adamus G Webb S Shiraga S Duvoisin RM Anti-recoverin antibodies induce an increase in intracellular calcium, leading to apoptosis in retinal cells. J Autoimmun 2006 26 146 53 16426815 10.1016/j.jaut.2005.11.007

[31] Heckenlively JR Solish AM Chant SM Meyers-Elliott RH Autoimmunity in hereditary retinal degenerations. II. Clinical studies: antiretinal antibodies and fluorescein angiogram findings. Br J Ophthalmol 1985 69 758 64 4052361 10.1136/bjo.69.10.758 PMC1040734

[32] Miyamoto K Khosrof S Bursell SE Rohan R Murata T Clermont AC Aiello LP Ogura Y Adamis AP Prevention of leukostasis and vascular leakage in streptozotocin-induced diabetic retinopathy via intercellular adhesion molecule-1 inhibition. Proc Natl Acad Sci USA 1999 96 10836 41 10485912 10.1073/pnas.96.19.10836 PMC17969

[33] Lewis GP Fisher SK Up-regulation of glial fibrillary acidic protein in response to retinal injury: its potential role in glial remodeling and a comparison to vimentin expression. Int Rev Cytol 2003 230 263 90 14692684 10.1016/s0074-7696(03)30005-1

[34] Cao X Bishop RJ Forooghian F Cho Y Fariss RN Chan CC Autoimmune retinopathy in systemic lupus erythematosus: histopathologic features. Open Ophthalmol J 2009 3 20 5 19554211 10.2174/1874364100903010020 PMC2701269

[35] Kerr EC Copland DA Dick AD Nicholson LB The dynamics of leukocyte infiltration in experimental autoimmune uveoretinitis. Prog Retin Eye Res 2008 27 527 35 18723108 10.1016/j.preteyeres.2008.07.001

[36] Carroll MC The complement system in regulation of adaptive immunity. Nat Immunol 2004 5 981 6 15454921 10.1038/ni1113

